# Vicarious Menstruation

**Published:** 1850-01

**Authors:** 


### OBSTETRICS.

### *Vicarious Menstruation.*

[Communicated to a distinguished Medical Gentlemen in Philadelphia.]

Dear Sir:—Yesterday, quite an interesting, and, so far as my knowledge extends, somewhat unique case was presented to me for medical advice. The following is a brief summary of the case, as detailed to me by the individual herself.

Miss M., æt. 20, of medium size, and dark complexion, was attacked with severe pains in the uterine region, about one year or more ago. She labored under a severe spell of sickness, under the care of Dr. Atlee and other physicians of Lancaster. For some time her life was despaired of, but she ultimately recovered, and for more than nine months subsequently was unable to *urinate*, save by the aid of the catheter. Her catamenial discharge appears monthly, and, according to her account, plentifully. Her menstruation continues usually about four days; but the most interesting fact is, that, at each menstrual period, she spits up large coagula of blood, and there is also a discharge from one of her nipples of a sanguineous fluid, both commencing, continuing and stopping at the same time with that of the menses. Now I have heard of vicarious menstruation, but then there was no discharge from the uterus at the same time, or, if any, it was very scanty. Can it be owing to an engorgement of the stomach and mammæ at the same time with that of the uterus? and if so, whence the cause? Would it be dangerous to attempt arresting these periodical flows from the stomach and mammæ? And if so, why should it be dangerous, inasmuch as a goodly discharge is going on from the uterus at the same time? The young woman is not any ways emaciated, but, on the contrary, looks quite plump, or as the French would say, *embonpoint.* She is troubled with considerable pain in the small of her back. Time will not allow me to say more at present. I wish you to answer this immediately, and give me your opinion on the subject, treatment, &c.

Yours respectfully, W. R. B.

[*Boston Medical Journal.*

